# Epidemiological and clinical characteristics of headache among medical students in Palestine: a cross sectional study

**DOI:** 10.1186/s12883-021-02526-9

**Published:** 2022-01-03

**Authors:** Firas Anaya, Wala’a Abu Alia, Feda’a Hamoudeh, Zaher Nazzal, Beesan Maraqa

**Affiliations:** 1 Faculty of Medicine and Health Sciences, An-Najah National University, Nablus, Palestine; 2 Primary Health Directorate, Ministry of Health, Ramallah, Palestine

**Keywords:** Tension- type headache, Migraine, Medical schools, Stress, Palestine

## Abstract

**Background:**

Headache is one of the most disturbing complaints worldwide, negatively impacting social and regular life activities. In the background of stressful life in medical schools, adding to the stressful situation in Palestine, a developing country under occupation, this study investigated the prevalence and clinical characteristics of migraines and tension- type headaches among medical students from the Palestinian Universities in West Bank and Gaza.

**Methods:**

A questionnaire-based cross-sectional study was conducted on all Palestinian Medical Students. Students were diagnosed based on ICHD-3 criteria. Demographic characteristics were compared by gender for each type of headache. Frequency, percentage, and mean ± SD. Pearson’s chi-squared test, independent t-test, and one-way ANOVA were used where needed. *P*-value < 0.05 was considered significant.

**Results:**

The study included 806 medical students; 476 (59.0%) of them were female. TTH and migraine’s prevalence was 59.8 and 22%, respectively, with a higher prevalence among basic year students. The female to male ratio was 1:0.6 for both types of headaches. Sleep deprivation, physical activities, and altered sleep patterns were reported as the top triggering factors.

**Conclusions:**

The results demonstrate that the prevalence of both subtypes’ primary headache is high among Palestinian medical students, with a higher prevalence among basic year students.

The study also showed that these findings are higher than other studies among medical students in other countries.

## Introduction

According to estimates, 50% of adults experience at least one headache per year [1]. At students’ level, headache interferes with their academic, social, and personal lives [2]. Medical students, in particular, have been the focus of attention in the previous headache studies [3–5], especially with the enormous academic load and stress they encounter during medical school [6].

This study aims to evaluate the prevalence and clinical characteristics of primary headaches (Migraine and Tension-type headache (TTH)) in all medical schools in Palestine, both in Gaza and West Bank, and the relieving methods used by medical students to alleviate their suffering.

## Subjects and methods

### Study design and population

This study utilized a cross-sectional design and took place between March 25 and June 25, 2019. It drew students from all Palestinian universities with medical schools located in the West Bank and Gaza. The Institutional Review Board approved the study.

### Data collection

The authors collected data using an Arabic-language self-administered questionnaire. The questionnaire was designed in accordance with the third edition of the International Classification of Headache version 3. (ICHD-3) [7]. It consisted of 28 items divided into six sections, and the questions varied between closed- and open-ended questions, Likert scales (Always, usually, often, sometimes & never), and visual analog.

The first section included demographic data: age, gender, medical year, marital status, university, self-reported academic performance (excellent, very good, good), and self-reported weight and height. Next, BMI was calculated and classified using WHO criteria; defined as a person’s weight in kilograms divided by the square of the person’s height in metres (kg/m2) and categorized to underweight, normal, overweight and obese [8].

The questionnaire assessed if students had any headache attack in their lifetime, medical school time, past year, and past 3 months in the second section depending on the second criterion for migraine “untreated or unsuccessfully treated Headache attacks lasting 4-72 hr” and the first criterion for TTH “ At least 10 episodes of headache fulfilling the ICHD-3 criteria for TTH”. In the third section, it assessed the most approximate frequency (number of attacks per day, days per week, weeks per month, months per year), approximate attack duration in hours (drop list with 0.5, 1, 2…24 h); depending on the second criterion for both migraine and TTH. Furthermore, it assessed the site, character, prodromal symptoms, and aura (visual disturbances or hallucinations, abnormal sensations, speech difficulty, language abnormalities, weakness, vertigo, or tinnitus): depending on the third and fourth criteria for both migraine and TTH. Family history (yes/no/unknown), and intensity which was represented as a visual analog scale and the scores converted later to levels (mild (0–3), moderate (4–6), severe (7–10)); depending on the third criterion for both migraine and TTH.

The fourth section addressed treatment and prevention strategies and a Likert scale (always, quite often, seldom, never) representing the students’ preferred pain relief methods (sleep, caffeine, medications, shower, specific herbs, specific food). The fifth section discusses possible precipitating factors (sleep deprivation, stress, long study hours, physical activities, altered sleep pattern, exams, social events, specific sounds, specific smells, hunger, food, weather, colorful lights, menses, caffeine, financial difficulties, smoking, energy drinks). Finally, in the sixth section, students were asked how headache affects their daily life activities (study, routine life, hobbies, sleep, social life, and exam delay), which were also represented using a Likert scale.

The questionnaire provides a detailed review of all aspects required for the differential diagnosis of headache according to ICHD-3 [7]. Two neurologists and one internist reviewed the questionnaire’s face and content to ensure that it contained all necessary components for diagnosis. Finally, the questionnaire was piloted with 30 medical students and revised as necessary.

**Fig. 1:**
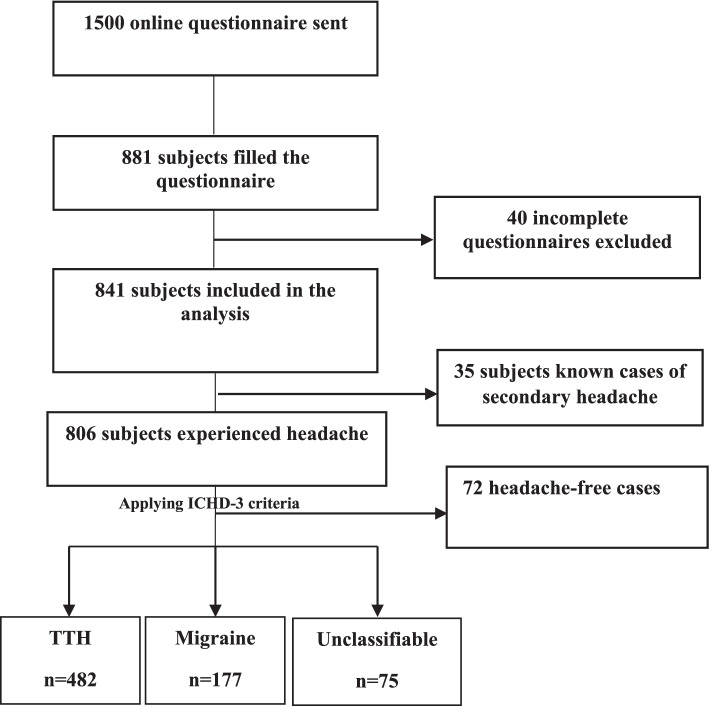
The flowchart of participants

The questionnaire was created online using Survey Monkey. On the first page of the questionnaire, informed consent has been requested. The questionnaire was distributed to students who consented. The link was emailed to all 1500 Palestinian medical students; 881 responded, a response rate of 58.7%. The proportion of respondents at each university is proportional to the number of medical students and the gender ratio at each university (see Fig. 1).

### Diagnosis

The authors used ICHD-3 criteria to diagnose students on an individual basis. Based on ICHD-3 criteria, the authors classified the diagnosis as Migraine or TTH. Others had no headaches and “Unclassifiable Headache,” which meant that none of the ICHD-3 criteria could be applied.

### Statistical analysis

The study used IBM SPSS Statistics for Windows, version 21 to analyze the data (IBM Corp., Armonk, NY, USA). The descriptive section included frequency, percentage, and mean standard deviation. To determine the distribution of students with headaches by gender and headache characteristics, the authors used Pearson’s chi-squared test, an independent t-test, and one-way ANOVA as needed. *P*-values less than 0.05 were considered statistically significant.

## Results

### Characteristics of the participants

A total of 881 students consented to the questionnaire; 40 incomplete questionnaires were excluded from further study, alongside another 35 questionnaires from students previously diagnosed with secondary headache (15 sinusitis, 10 TMJ, nine vision problems, one pseudotumor cerebri). The students’ net number was 806, with a female-to-male ratio of 1.4:1 proportional to the female-male rate in all Palestinian medical schools. The number of participants from each university is also proportional to each university’s overall number in general and, specifically, medical students in that university. Table 1 shows the demographic characteristics of participants included in the study.

**Table 1:** Demographic characteristics of participants included in the study (*n* = 806)

	Total (%)	Male ***N*** = 330 (41%)	Female ***N*** = 476 (59%)
***Age,*** *mean ± SD (range)*	21 ± 2 (9)	21 ± 2 (9)	21 ± 2 (9)
***Marital Status***			
Single	771 (95.6)	323 (97.8)	448 (94.1)
Married	35 (04.3)	7 (02.1)	28 (05.8)
***University***			
An-Najah National university	286 (35.4)	121 (36.6)	165 (34.6)
Al-Quds	201 (24.9)	69 (20.9)	132 (27.7)
Al-Islamiyah	169 (20.9)	77 (23.3)	92 (19.3)
Al-Azhar	150 (18.6)	63 (19.0)	87 (18.2)
***Year***			
Basic	419 (51.9)	159 (48.1)	260 (54.6)
Clinical	387 (48.0)	171 (21.2)	216 (45.3)
***Performance***			
Excellent	144 (17.8)	57 (17.2)	87 (18.2)
Very Good	416 (51.6)	175 (53.0)	241 (50.6)
Good	246 (30.5)	98 (29.6)	148 (31.0)
***BMI***			
Underweight	66 (08.1)	16 (04.8)	50 (10.5)
Normal	522 (64.7)	182 (55.1)	340 (71.4)
Overweight	178 (22.0)	107 (32.4)	71 (14.9)
Obese	40 (04.9)	25 (07.5)	15 (03.1)

### The prevalence of headache by gender

For the life-time prevalence of headache, 482 (59.8%; [95%CI: 56.1–62.9%]) students [55.8% of males vs 62.6% females (*P*-value 0.051)] were diagnosed with TTH, while 177 (21.9%; [95%CI: 19.2–25.0%]) students [19.4% of males vs 23.7% of females (P-value 0.141)] were diagnosed with migraine based on ICHD-3 criteria. The overall lifetime prevalence of primary headache (i.e., TTH & Migraine) was 81.8% [95%CI: 78.9–84.3%]; significantly higher among females (86.3%) compared to males (75.2%) (*P*-value < 0.001).

Results showed that 273 (33.9% [95%CI: 30.6–37.3%) students have TTH in the last year [29.4% of males vs 37.0% of females (*P*-value 0.025)] while it was 116 (14.4%) [21.1% of males vs 16.0% of females (*P*-value 0.126) for migraine. 48.3% [95%CI: 45.9–52.9%) of the students had primary headache in the last year; 41.5% of males compared to 52.9% of females (*P*-value 0.001) (Table 2).

**Table 2:** The distribution of lifetime and last year prevalence of headache’s types with gender

	All n (%)	Male n (%)	Female n (%)	***P***-value	OR (95%CI)
**Life-Time**					
*TTH*	482 (59.8)	184 (55.8)	298 (62.6)	0.051	0.89 (0.79–1.0)
*Migraine*	177 (22)	64 (19.4)	113 (23.7)	0.143	0.81 (0.62–1.1)
*Unclassifiable*	75 (9.3)	44 (13.3)	31 (6.5)	0.001	2.0 (1.3–3.1)
*Free*	72 (8.9)	38 (11.5)	34 (7.1)	0.032	1.6 (1.03–2.5)
*Primary*	659 (81.8)	248 (75.2)	411 (86.3)	< 0.001	0.87 (0.81–0.93)
**Last Year**					
*TTH*	273 (33.9)	97 (29.4)	176 (37)	0.025	0.79 (0.64–0.97)
*Migraine*	116 (14.4)	40 (21.1)	76 (16)	0.126	0.75 (0.53–1.1)
*Unclassifiable*	278 (34.5)	114 (34.5)	164 (34.5)	0.979	1.0 (.82–1.2)
*Free*	139 (17.2)	79 (23.9)	60 (12.6)	< 0.001	1.8 (1.4–2.6)
*Primary*	398 (48.3)	137 (41.5)	252 (52.9)	0.001	0.78 (0.67–0.91)

### The prevalence of headache by demographics

The study’s findings demonstrate that both the academic year and the university are related to the prevalence of TTH. TTH was most prevalent in clinical years (*p* = 0.017). Additionally, students at An-Najah university had the highest prevalence of TTH (65.8%) and the lowest prevalence of migraines. (0.02; *p* = 0.02) (Table 3).

**Table 3:** The prevalence of lifetime headache by age, sex, university, academic year, and performance

Variable	Migraine			TTH		
	Yes n (%)	NO n (%)	P-Value	Yes n (%)	NO n (%)	***P***-Value
***Age,*** *mean ± SD*	21.1 ± 2.1	20.9 ± 2.1	0.492	21.1 ± 2.1	20.8 ± 2.1	0.027
***Sex***						
Males	64 (36.1)	266 (42.2)	0.143	184 (38.1)	146 (45)	0.051
Female	113 (63.8)	363 (57.7)		298 (61.8)	178 (54.9)	
***Marital Status***						
Single	166 (93.7)	605 (96.8)	0.167	460 (95.4)	311 (95.9)	0.706
Married	11 (06.2)	24 (3.8)		22 (4.5)	13 (4)	
***University***						
An-Najah	52 (29.3)	234 (37.2)	0.083	196 (40.6)	90 (27.2)	0.002
Al-Quds	41 (23.1)	160 (25.4)		114 (23.6)	87 (26.8)	
Al-Islamiyah	42 (23.7)	127 (20.1)		90 (18.6)	79 (24.3)	
Al-Azhar	42 (23.7)	108 (17.1)		82 (17)	68 (20.9)	
***Year***						
Basic	92 (51.9)	327 (51.9)	0.998	234 (48.5)	185 (57.5)	0.017
Clinical	85 (48.1)	302 (48.1)		248 (51.4)	139 (42.9)	
***Academic Performance***						
Excellent	30 (16.9)	114 (18.1)	0.163	87 (18)	57 (17.5)	0.945
Very Good	102 (57.6)	314 (49.9)		250 (51.8)	166 (51.2)	
Good	45 (25.4)	201 (31.9)		145 (30)	101 (31.1)	
***Family History***						
Yes	87 (49.1)	218 (34.6)	< 0.001	192 (39.8)	113 (34.8)	< 0.001
NO	55 (31.1)	206 (32.7)		175 (36.3)	86 (26.5)	
Unknown	35 (19.7)	205 (32.5)		115 (23.8)	125 (38.5)	

### Headache characteristics

The study shows that the mean duration of a migraine attack was 6.0 ± 4.6 h, significantly higher among females than male students (*p* = 0.019). The mean duration for the TTH attack was 3.2 ± 2.7 h; there was no significant difference between male and female students (*p* = 0.059). The frequency of headache attacks per year for Migraine was 46.5 ± 61.2 (males: 41.7 ± 60.1, females: 49.2 ± 61.8), while 57.4 ± 69.6 (males: 49.3 ± 63.5, females: 62.2 ± 72.7) for TTH and 2.6 ± 3.4 (males: 2.2 ± 2, females: 3.1 ± 4.7) for Unclassifiable headache. Half of the students (50.2%) with Migraines and 63.0% of students with TTH experienced moderate attacks (Table 4).

**Table 4:** Students’ migraine, TTH, and unclassifiable headaches characteristics with gender

		Male n (%)	Female n (%)	Total n (%)	P-value
**Migraine**	***Duration*** *mean ± SD*	5.3 ± 4.4	6.5 ± 4.6	6.0 ± 4.6	0.019
	***Episode Number*** *mean ± SD*	41.7 ± 60.1	49.2 ± 61.9	46.50	0.268
	***Severity level***				
	Mild	1 (1.5)	22 (19.4)	23 (12.9)	0.053
	Moderate	41 (64)	48 (42.4)	89 (50.2)	
	Severe	22 (34.3)	43 (38)	65 (36.7)	
**THH**	***Duration*** *mean ± SD*	2.9 ± 2.1	3.3 ± 3.0	3.2 ± 2.7	0.059
	***Episode Number*** *mean ± SD*	49.3 ± 63	62.2 ± 72	57.4 ± 69	0.008
	***Severity level***				
	Mild	41 (22.2)	47 (15.7)	88 (18.2)	0.052
	Moderate	112 (60.8)	192 (64.4)	304 (63.0)	
	Severe	31 (16.8)	59 (19.7)	90 (18.6)	
**Unclassified headache**	***Duration*** *mean ± SD*	2.3 ± 0.8	2.3 ± 1.3	2.3 ± 1.0	0.937
	***Episode Number*** *mean ± SD*	2.2 ± 2.1	3.2 ± 4.7	2.6 ± 3.5	0.131
	***Headache intensity***				
	Mild	9 (20.4)	3 (9.6)	12 (16.0)	0.861
	Moderate	22 (50.1)	20 (64.5)	42 (56.0)	
	Severe	13 (29.5)	8 (25.8)	21 (28.0)	

### Triggering factors

Sleep deprivation appeared to be a primary trigger for both migraines and TTH. Additional critical factors include physical activity, stress, and long study hours (Table 5).

**Table 5:** Distribution of Migraine and TTH triggering factors

Triggering Factors	Migraine	Rank	TTH	Rank
***Sleep Deprivation***	89.8%	1	88.5%	1
***Physical activities***	75.7%	3	82.7%	2
***Stress***	70.0%	4	81.1%	3
***Long Study hours***	70.0%	4	79.8%	4
***Altered Sleep Pattern***	80.2%	2	76.3%	5
***Exam’s Stress***	66.1%	5	75.7%	6
***Social Events***	57.0%	8	58.7%	7
***Hunger***	59.8%	7	58.0%	8
***Noise***	61.5%	6	56.6%	9
***Menstruation***	38.5%	11	55.7%	10
***Specific Weather***	53.6%	10	47.0%	11
***Colorful lights***	54.8%	9	43.1%	12
***Caffeine consumption***	32.7%	13	37.7%	13
***Money***	32.7%	14	35.6%	14
***Odors***	38.4%	12	27.3%	15
***Smoking***	19.2%	15	20.9%	16
***Energy Drinks***	13.5%	17	17.0%	17
***Specific Food***	19.2%	16	16.1%	18

### Relieving methods during an attack

**Fig. 2:**
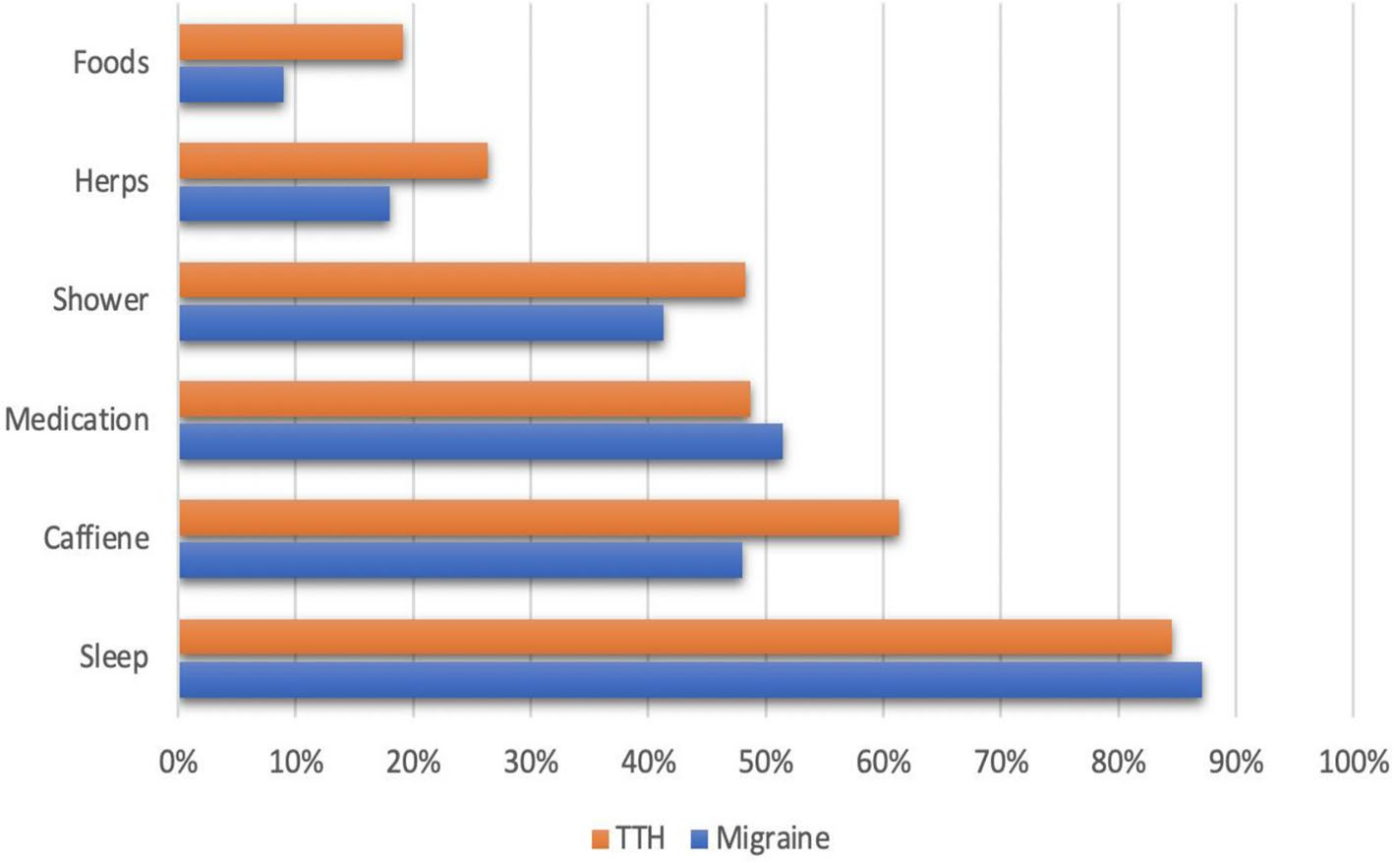
Methods alleviate students suffering from Migraine and TTH

The majority of students with migraines (87.1%) reported sleeping to alleviate their suffering, followed by medication (51.5%)—other methods, as seen in Fig. 2.

## Discussion

Several contributing factors, including stress, anxiety, and depression, appear early in medical school [9, 10]. Research has shown that stress can cause headaches [11]. A broad community-based study indicates that college students are at higher risk of a headache than the general population [12]. This research aims to establish the prevalence and clinical characteristics of both migraine and TTH among medical schools in Palestine.

The overall lifetime prevalence of primary headache (i.e., TTH & Migraine) was 81.8%. In detail, 482 (59.8%) [184 (55.8%) males and 298 (62.6%) females] and 177 (22.0%) patients, respectively, were diagnosed with TTH and Migraine. Females have a higher prevalence of migraines (23.7%) than males (19.4%). This corresponds to females’ greater global dominance, which is shown to be two to three times as common in females as it is in males [13]. Females also have a higher prevalence of TTH in our study. This prevalence was higher than those conducted in other developing countries; 46.0% in Nigeria, [14] 58.7% in Iran, [15] and 40.0% in Brazil [16]. Some countries showed an almost similar high lifetime prevalence of primary headaches, like Pakistan (87.8%) [17]. This raises concerns about this burden among the Palestinian population and particularly among the medical students in Palestine. As an occupied territory, Palestine is faced with a unique focus of the ongoing Israeli occupation. Medical students cannot return to their homes every day due to Israeli checkpoints and cross-border travel. They must live alone with colleagues in some apartments that lack several basic amenities, contributing to an increase in tension and potential psychosocial strain.

For various forms of headache, TTH was more prevalent in this research than migraine, which is consistent with results from another study in Iran [15]. When compared to other studies conducted on medical students, it was higher than the prevalence in Nigeria (18.1%), [14] Iran (44.2%), [15] and Nairobi (50.0%) [18]. In Brazil, they reported a lower prevalence of TTH (75.7%) compared to migraine [16]. In comparison, migraine prevalence in this study was lower than in other studies, [3, 18] yet higher than the majority in Soochow (7.9%), [19] Iran (14.2%), [15] Nigeria (6.4%), [14] and Turkey (12.6%) [20]. Even though differences in methodology or participation proportion can explain these differences, they are still very high.

Our findings indicate that TTH is significantly higher in clinical years than in primary years. This could be because clinical years are stressful, with extended study hours, higher passing scores, and frequent and interrupted travel between clinical sites.

This study shows a 49.1% positive family history among migraine cases, which is higher than the association reported in previous studies, such as those conducted in India (31.0%) and Nigeria (22.0%) [14, 21]. The age-standardized average for headaches in Palestine is 16,702 per 10,000 and 32,495 per 100,000 for migraine and TTH, respectively, with the highest percentage shift in counts in the EMR region after Pakistan [22]. Higher rates of depression and anxiety play a significant role in Palestine than the rest of the world [23]. Stressful life events such as persistent conflict can also lead to a higher burden of headaches. Despite that family history was self-reported, this high percentage and the elevated age-standerized average for headaches encourage us to implement a screening program for the Palestinian population.

In the present study, most migraine students suffered from moderate to severe headaches, consistent with other studies [3, 24]. At the same time, most of the TTH students suffered from a moderate headache. This should urge the universities’ officials to implement relieving strategies among this group and guidance about stress-relieving methods.

As shown in this study, females have a higher prevalence of TTH and tend to have more frequent episodes throughout their lives, consistent with literature [22]. The nature of both genders could explain this difference and hormonal differences. In addition to this, lack of sleep was the most frequent trigger of both TTH and Migraine, consistent with the findings of studies conducted in China [19] and Kuwait [3]. This is a sensible finding because of the considerable study load and limited study hours during med school. Students here can be guided by their seniors on how to organize their study schedule during the day in a way that secures enough sleeping hours for them.

This study is one of few studies focusing on both Migraine and TTH in medical students, and it is the first study covering all medical schools in Palestine. In addition to the recall bias, the low participation proportion dictated by using email for data collection is the main limitation of this study. Since the West Bank and Gaza are under Israeli military control, the email was chosen because of restricted alternatives. Limited cooperation between Palestinian universities, on the other hand, restricts our ability to train data collectors at each university. The online form was supposed to be comfortable and user-friendly. Still, many students who erroneously exit the document could not re-enter the form because we restricted the form’s usability to only one time per computer to prevent multiple responses, and their responses were considered incomplete. Additionally, this study makes no mention of the impact of headache on the student’s life, which should be addressed in further studies. On the other hand, when it comes to Palestinian population, we documented headaches with various diagnoses in medical students in an environment where there is a paucity of research on Palestinian students’ life interactions and associated morbidities.. Furthermore, certain variables, particularly those relating to alleviating techniques, got little consideration in our study. Therefore, we propose future research in this area.

## Conclusion

In conclusion, this study assessed the prevalence of both Migraine and TTH and its characteristics among Palestinian medical students and found it very high compared to many previous studies among Medical students. Since these results can’t be generalized, further research should be conducted to determine the prevalence of headaches and their subtypes among the Palestinian population and all university students to compare them with the medical students. A validation study for the questionnaire is recommended to be used for clinical and research purposes.

## Data Availability

The data used to support the findings of this study are available from the corresponding author upon request.
